# A Single Fluorescent Protein-Based Indicator with a Time-Resolved Fluorescence Readout for Precise pH Measurements in the Alkaline Range

**DOI:** 10.3390/ijms232112907

**Published:** 2022-10-26

**Authors:** Tatiana R. Simonyan, Elena A. Protasova, Anastasia V. Mamontova, Aleksander M. Shakhov, Konstantin A. Lukyanov, Eugene G. Maksimov, Alexey M. Bogdanov

**Affiliations:** 1 Center of Molecular and Cellular Biology, Skolkovo Institute of Science and Technology, 121205 Moscow, Russia; 2 Shemyakin-Ovchinnikov Institute of Bioorganic Chemistry, 117997 Moscow, Russia; 3 Faculty of Biology, M.V. Lomonosov Moscow State University, 119992 Moscow, Russia; 4 Semenov Federal Research Center for Chemical Physics, 119991 Moscow, Russia

**Keywords:** fluorescent indicators, time-resolved spectroscopy, fluorescence lifetime, FLIM, pH

## Abstract

The real-time monitoring of the intracellular pH in live cells with high precision represents an important methodological challenge. Although genetically encoded fluorescent indicators can be considered as a probe of choice for such measurements, they are hindered mostly by the inability to determine an absolute pH value and/or a narrow dynamic range of the signal, making them inefficient for recording the small pH changes that typically occur within cellular organelles. Here, we study the pH sensitivity of a green-fluorescence-protein (GFP)-based emitter (EGFP-Y145L/S205V) with the alkaline-shifted chromophore’s pKa and demonstrate that, in the pH range of 7.5–9.0, its fluorescence lifetime changes by a factor of ~3.5 in a quasi-linear manner in mammalian cells. Considering the relatively strong lifetime response in a narrow pH range, we proposed the mitochondria, which are known to have a weakly alkaline milieu, as a target for live-cell pH measurements. Using fluorescence lifetime imaging microscopy (FLIM) to visualize the HEK293T cells expressing mitochondrially targeted EGFP-Y145L/S205V, we succeeded in determining the absolute pH value of the mitochondria and recorded the ETC-uncoupler-stimulated pH shift with a precision of 0.1 unit. We thus show that a single GFP with alkaline-shifted pKa can act as a high-precision indicator that can be used in a specific pH range.

## 1. Introduction

Among the existing approaches to pH measurement, a special place is occupied by the optical techniques, whose development, implementation and improvement has been described across a whole galaxy of scientific papers [1]. In biological systems, pH acts as a critical parameter characterizing the state of the body fluids, tissues and cells, as well as their homeostasis and physiological functionality [2,3,4,5,6,7]. Inside the cell and subcellular structures, even small pH shifts can transmit important biochemical messages, mediating processes such as a change in the cell polarity [8,9], the reorganization of the cytoskeleton [10,11] and cell migration [8,9,12,13]. The precise measurement of the intracellular pH undoubtedly represents a technical challenge. Based on general considerations, the probe of choice for biological pH-metry must comply with several requirements. Thus, it must (i) possess a sufficient sensitivity within the relatively narrow pH range of ~5–8, relevant for the cellular organelles; (ii) provide the determination of an absolute pH value with an accuracy of around 0.1 unit; and (iii) be minimally invasive with respect to the live cell physiology. Genetically encoded fluorescent pH indicators (often based on the fluorescent protein (FP) variants) effectively satisfy the third specified demand [14,15,16,17,18]. In most cases, however, the imaging of such probes relies on the fluorescence intensity readout and, thus, enables the monitoring of the relative pH changes but not the determination of the absolute pH value [19,20]. To enable quantitative-fluorescence-based pH analysis, one may use some intrinsic characteristic of the pH-sensitive fluorophore as a signal readout. In particular, several examples in which the fluorescence decay kinetics of FP-based genetically encoded probes were found to be sensitive to the intracellular pH and could be visualized using fluorescence lifetime imaging microscopy (FLIM) have been described [21,22,23,24,25,26]. Being determined by the excited state relaxation rates of a particular fluorophore in a specific physicochemical environment, the fluorescence lifetime is weakly affected by the peculiarities of the experimental setup, such as the protein expression level, detector settings and excitation/emission filter specifications [27], making it advantageous for quantitative analysis. Existing genetically encoded lifetime-based pH probes are most affected by the modest dynamic range (DR) of their signal and/or short range of the pH sensitivity, which rarely covers the whole diversity of pH values naturally occurring in the cellular compartments. Thus, the red-emitting pHRed showed a lifetime change of 0.4 ns in the pH range of 6.0–8.0 [24], while for the cyan ECFP, a 0.8 ns shift (which corresponded to the DR of 32%) within the pH range of 5.0–7.0 was documented [21]. The variants possessing a lifetime change of ≥1 ns, such as pH-Lemon [25] and SypHer3s/SypHerExtra [26], demonstrated a well-marked drift of their pH sensitivity towards the acidic and alkaline ranges, respectively. Finally, the green-emitting EGFP-pHsens, which showed sensitivity to pH changes in a relatively wide range of 5–8, was characterized by multicomponent fluorescence decay kinetics, with small changes in the components’ lifetimes and amplitudes [23]. Hence, the attractive concept of a universal fluorescent pH meter combining a wide range of pH sensitivities and a high DR is probably not able to be implemented in the case of any of the listed indicators. Moreover, even a theoretical probe optimized to obtain an enhanced DR and sensitivity range may show insufficiently low signals for measurements in the local pH range and is unlikely to have a monotonic lifetime/pH dependence over the entire measurement range.

As an alternative approach, one can consider the idea of designing/improving separate, specialized lifetime probes covering several narrow ranges of intracellular acidity and working in a way that is complementary to the pH papers (e.g., the pH-Lemon, which shows an excellent performance in the acidic compartments [25]). In this case, one could expect that the local pH sensitivity of an FP-based indicator is observed near the pKa value of its chromophore. Here, we test a variant of the enhanced green fluorescent protein (EGFP [28]) with a strong alkaline pKa shift—EGFP-Y145L/S205V—as a prospective pH indicator. As we reported earlier, the mutations introduced to the nearest chromophore environment of EGFP-Y145L/S205V result in chromophore protonation at a neutral pH and the partial inhibition of the excited state proton transfer (ESPT) chain and switch its fluorescence decay to the multicomponent mode [29]. We understand that these factors are prerequisites for a high pH sensitivity throughout the fluorescence lifetime in the mild alkaline range based on the differential characteristics of the fluorescence emission from the neutral and anionic chromophore.

## 2. Results and Discussion

### 2.1. The pH Sensitivity of the Purified Protein

**Figure 1:**
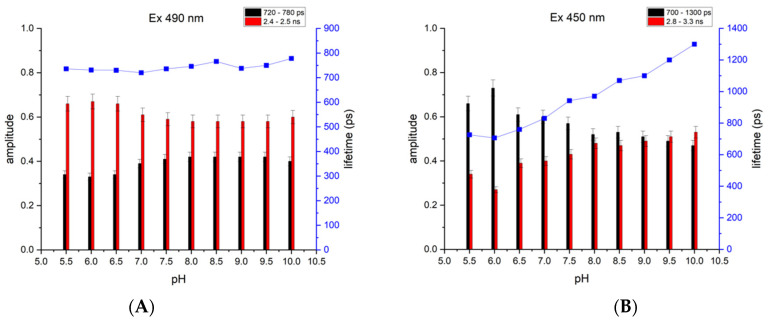
Relative contributions (relative amplitude, left vertical axis) and lifetimes (the value of the short-lived component is shown as blue squares, right vertical axis) of both components based on the biexponential fit of the fluorescence decay data of EGFP-Y145L/S205V upon excitation with (**A**) a 490 nm, single-photon laser source and (**B**) a 450 nm, single-photon laser source at different pH levels varying from pH = 5.5 to pH = 10.0. The black columns denote the contribution of the short-lived component, and the red columns denote the long-lived component. The blue squares and the solid line (right axis) show the lifetime of the short-lived component.

Earlier, we showed that at the neutral pH level, EGFP-Y145L/S205V demonstrated biphasic fluorescence decay upon both single- and two-photon fluorescence excitation, targeted towards the anionic chromophore state (490/980 nm). We supposed that the components of this decay, which had similar photon contributions and the lifetimes of ~0.7 and ~2 ns, correspond to the emission of neutral (λ_ex_ ~ 400 nm, λ_em_ ~ 460 nm) and anionic (λ_ex_ ~ 490 nm, λ_em_ ~ 510 nm) chromophores, respectively [29]. If this is correct, then the primary examination of the EGFP-Y145L/S205V as a pH indicator should enable the determination of the fluorescence excitation mode, which provides a better expression of the lifetime’s sensitivity to the pH. To this end, we used available single-photon light sources (a picosecond laser, λ_em_ = 450 nm, and a femtosecond laser, λ_em_ = 490 nm) to measure the fluorescence decay kinetics of the protein samples, represented by the purified EGFP-Y145L/S205V dissolved in the buffers with the pH values of 5.5–10.0 (Figure 1, Supplementary Figures S2 and S3, Supplementary Table S1). In both cases, the decay data were satisfactorily fitted using a two-exponential model. Upon excitation at 490 nm (absorbed mostly by an anionic chromophore), the lifetimes of both components of the fluorescence emission from EGFP-Y145L/S205V were found to be almost insensitive to the pH, while those detected upon excitation at 450 nm (absorbed by neutral and anionic chromophores) showed a moderate, albeit visible, pH response in the 6.5–9.5 range (Supplementary Figure S1). In particular, the shorter component’s lifetime (which we can attribute to a blue emission from the neutral GFP chromophore) rises almost twofold (from 700 to 1300 ps) in the pH range of 5.5–10.0.

**Figure 2:**
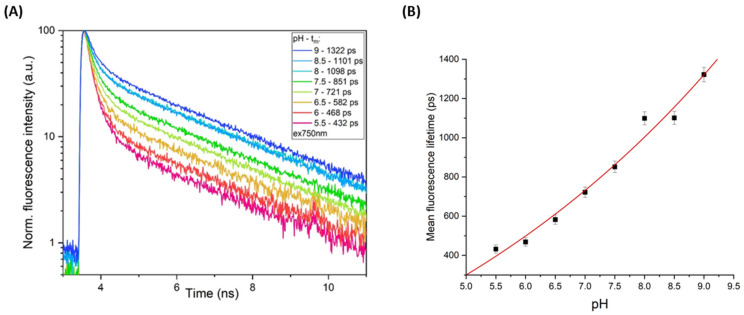
The pH-dependent photobehavior of EGFP-Y145L/S205V recorded by time-resolved fluorescence spectroscopy upon two-photon 750 nm excitation. (**A**) Normalized fluorescence decay kinetics at different pH levels, varying from pH = 5.5 to pH = 9.0. The mean lifetime values and the color legend are in the inset in the top right area. (**B**) The graph describing the dependence of the mean lifetime on the pH.

One can thus suppose that the further shift of the fluorescence excitation towards the shortwave range, where the neutral chromophore is predominantly responsible for the absorption, could provide better a visualization of the lifetime flexibility upon the pH shift. To test this assumption, we repeated the measurements of the purified EGFP-Y145L/S205V using a femtosecond two-photon light source emitting at 750 nm. While the fluorescence decay maintained its biexponential character, the lifetimes of the short-lived component decreased significantly (to τ_1_ ~250 ps, see Supplementary Figure S4). Importantly, both the emissive species detected upon 2P-750 nm excitation showed a pronounced pH-dependent behavior. Thus, the amplitude-normalized lifetime of the long-lived component (A_2_*τ_2_) underwent a 4-fold rise in response to the pH shift from 5.5 to 9.0 (Supplementary Figure S4). The mean lifetime (Τm) of the EGFP-Y145L/S205V emission also displayed a well-defined pH dependence and can probably be adequately used as a signal readout. Upon pH titration from 5.5 to 9.0, we recorded a 3-fold Τm rise (from ~400 to ~1300 ps, Figure 2A), and the data points on the Τm/pH graph exhibited a quasi-linear curve (Figure 2B), whose steep slope could provide a high-precision determination of the pH value.

### 2.2. The pH Dependence of the EGFP-Y145L/S205V Lifetime in Cellulo

**Figure 3:**
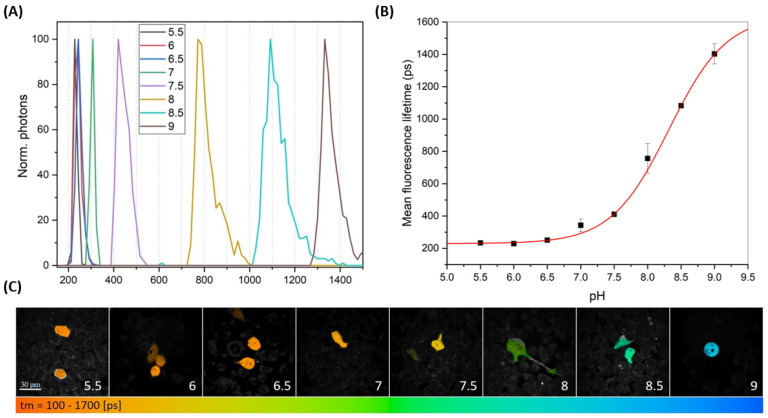
FLIM data recorded from the monensin/nigericin-penetrated HEK293T cells expressing EGFP-Y145L/S205V, pre-incubated with pH buffers (pH 5.5–9.0). (**A**) Histograms showing the mean lifetime distribution calculated from the FLIM scans at different pH values (color legend is in the inset). (**B**) The graph describing the dependence of the mean lifetime on the pH. (**C**) Color-coded FLIM scans exemplifying the lifetime values recorded in the cytoplasm of the HEK293T cells (color legend is under the image gallery). Scale bar is 30 µm. A two-photon (2P) laser, λ_em_ = 750 nm, was used for the fluorescence excitation.

Next, we prepared the construct for the mammalian expression of EGFP-Y145L/S205V, optimized the transfection conditions to provide a satisfactory fluorescence signal of this relatively dim probe in live HEK293T cells, and proceeded to conduct FLIM experiments using the femtosecond 2P-750 nm laser for the fluorescence excitation. Firstly, we focused on recording the pH dependence of the probe’s lifetime and its comparison with that recorded in the protein solution. To this end, we incubated live cells with an ionophore mixture prior to each imaging series to assess the permeability of the plasma membrane for the extracellular solution and replaced the culture medium with pH buffer (from 5.5 to 9.0, the same as that used for the protein specimens) to extrinsically set the intracellular pH value. FLIM scans taken at pH 5.5–6.5 displayed short (~250 ps) and weakly distinguishable mean lifetimes (Figure 3). The mean lifetime (Τm) at pH 7 was found to be slightly longer (~300 ps), albeit distinguishable from the neighboring points’ values. In the pH range of 7.5–9.0, there was a clearly resolved Τm distribution, with the highest relative resolution (from 400 to 800 ps) spotted between pH 7.5 and 8.0 (Figure 3). Importantly, a quasi-linear dependence of the Τm on the pH, with nearly monotonic (~300 ps) lifetime intervals between the data points, was observed in the pH range of 7.5–9.0 (Figure 3B).

In fact, the curve describing the pH responsiveness of the EGFP-Y145L/S205V lifetime (Figure 3B) can be used as a calibration plot for the signal quantification of this pH indicator. The reason why the linear region of the lifetime pH dependence in cellulo has an approximately 1.5 pH-unit alkaline shift compared to that recorded for the purified protein (see Figure 2B vs. Figure 3B) remained unclear. We suppose that such a phenomenology could be connected with either the difference between the apparent pKa values of EGFP-Y145L/S205V in the intracellular environment vs. the aqueous solution or with the incomplete equilibration of the pH between the extracellular and intracellular media, which could be a feature of the ionophore-penetration-based experimental setup. In any case, the EGFP-Y145L/S205V fluorescence response was characterized by a high relative amplitude, with the dynamic range of ~3.5 (~350%) within the apparent pH range of 7.5–9.0, thus showing potential for the highly accurate determination of the absolute pH value in the cellular compartments possessing a weak alkaline interior medium.

### 2.3. Static and Dynamic pH Measurements of the Mitochondria

**Figure 4:**
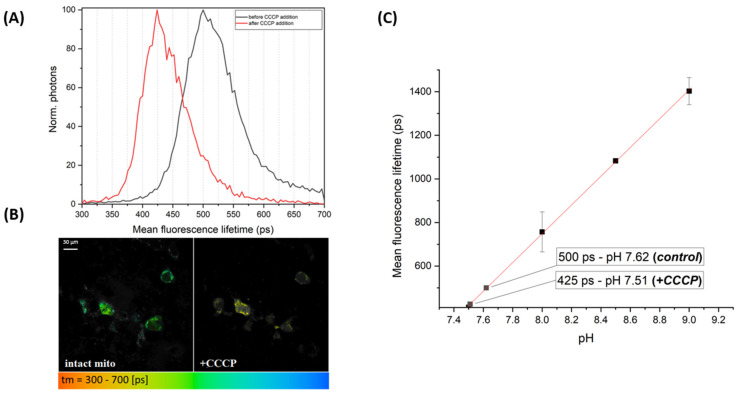
FLIM data recorded for the HEK293T cells expressing EGFP-Y145L/S205V in the mitochondria. (**A**) Histograms showing the distribution of the probe’s mean fluorescence lifetime in the intact mitochondria (black solid line) and after the addition of the ETC uncoupler (10 uM CCCP; red solid line). (**B**) Color-coded FLIM scans exemplifying the lifetime values recorded in the mitochondria of HEK293T cells before (right) and after (left) the addition of 10 uM CCCP (color legend is under the image gallery). Scale bar is 30 µm. A two-photon (2P) laser, λ_em_ = 750 nm, was used for the fluorescence excitation. (**C**) Calibration curve (part of the Tm/pH dependence measured in the cytoplasm) used to determine the absolute pH values of the mitochondria. The mean lifetime and corresponding pH values are shown in the boxes.

To assess the reliability of the Tm/pH calibration curve obtained for EGFP-Y145L/S205V in the previous experiments, we targeted the mitochondria, which are known to have a mild alkaline milieu [30,31], in the application of this probe and performed time-resolved fluorescence imaging of the live HEK293T cells to estimate the pH value in the matrix of their mitochondria by measuring the EGFP-Y145L/S205V fluorescence lifetime. FLIM images of the intact cells showed a mean fluorescence lifetime of ~500 ps (Figure 4), which corresponded to the pH value of ~7.62 (Figure 4C), which is consistent with the literature data [32]. This result proved the applicability of the calibration plot (Figure 3B and Figure 4B) and led us to reject the hypothesis of an unequal extra- and intracellular pH formed upon ionophore treatment. To verify the performance of the indicator under conditions stimulating a physiologically mediated pH shift in the mitochondria (i.e., to record the pH dynamics in live cells), we treated the cells with CCCP (carbonyl cyanide m-chlorophenyl hydrazone), an uncoupler of the mitochondrial electron transport chain, which inhibits oxidative phosphorylation through the uncoupling of the proton gradient in the inner mitochondrial membrane [33]. CCCP molecules spontaneously diffuse throughout the plasma membrane and enter the mitochondria, leading to a slight acidification of their matrix, which is well-described in the literature [33].

The FLIM visualization of the CCCP-stimulated live HEK293T cells displayed the mean lifetime shortening (to ~425 ps, Figure 4) of the indicator, which can be quantified as a decrease in the pH value to ~7.51 (Figure 4C). Importantly, the lifetime distribution histogram (Figure 4A) displayed clearly resolved signal peaks for the measurements of the intact and CCCP-stimulated cells, thus indicating the reliability of the pH value determination.

## 3. Materials and Methods

### 3.1. Protein Expression, Purification and Sample Preparation

The EGFP-Y145L/S205V coding sequence in the pQE30 vector backbone (Qiagen, Hilden, Germany), with an N-terminal 6xHis tag, was expressed in the *E. coli* XL1 Blue strain (Invitrogen, Waltham, MA, USA) and purified using TALON metal affinity resin (Clontech, Kyoto, Japan) according to the manufacturer’s protocol. For the further fluorescence spectroscopy experiments, the spherical resin particles with the immobilized proteins were washed four times with phosphate-buffered saline (PBS pH 7.4, Gibco, Waltham, MA, USA) at a ratio of 1:20 *v*/*v*. The proteins were then solubilized using 100 mM imidazole, pH 8.0, and desalted by ultrafiltration with Amicon^®^ Ultra-0.5 10 K (Merck, Tullagreen, Carrigtwohill Co Cork, Ireland) columns (the final buffer was PBS pH 7.4).

The time-resolved fluorescence spectroscopy of the purified protein was performed using a set of pH buffers in a pH range of 5.5–10.0. Each buffer contained 130 mM potassium gluconate, 20 mM sodium gluconate, 0.5 mM MgCl_2_, 0.2 mM EGTA and 30 mM Tris (pH 8.0–10.0), HEPES (pH 6.8–7.8) or MES (pH 5.5–6.6). Each specimen contained 5 μg/mL of EGFP-Y145L/S205V, dissolved in pH buffer at a ratio of 1:20 *v*/*v*.

### 3.2. Cloning

For the mammalian cell expression, PCR-amplified (forward with 5′-ATCGACCGGTGCCACCATGGTGAGCAAGGGCGAGG-3′ and reverse with 5′-ATCGGCGGCCGCTTACTTGTACAGCTCGTCCATGCC-3′) AgeI/NotI fragment encoding EGFP-Y145L/S205V was cloned in a pEGFP-N1 vector backbone (Clontech, Kyoto, Japan) instead of EGFP.

For the mitochondrial expression, PCR-amplified (forward with 5′-ATCGACCGGTCGCCACCATGGTGAGCAAGGGCGAGG-3′ and reverse with 5′-ATCGGCGGCCGCTTACTTGTACAGCTCGTCCATGCC-3′) AgeI/NotI fragment encoding EGFP-Y145L/S205V was cloned in a pTagRFP-mito vector backbone (Evrogen, Moscow, Russia) instead of TagRFP. The mitochondrial targeting sequence (MTS) was derived from the subunit VIII of human cytochrome C oxidase [34,35].

### 3.3. Fluorescence Lifetime Spectroscopy of the Aqueous Solution of the Purified Protein

Measurements were carried out using three different instrumental setups.

#### 3.3.1. Single-Photon 490 nm Fluorescence Excitation

Femtosecond laser pulses (80 MHz repetition rate, 100 fs, up to 25 nJ per pulse) from a Ti:Sapphire oscillator (Tsunami, Spectra-Physics, Andover, MA, USA) were applied at an 8 MHz repetition rate using an acousto-optic modulator (Pulse select, APE, Berlin, Germany) and a frequency doubled in the BBO SHG crystal. Then, femtosecond pulses with a central wavelength of 490 nm were coupled with an inverted optical microscope (IX71, Olympus, Tokyo, Japan) using a dichroic mirror (DMLP505, Thorlabs, Newton, MA, USA) and successfully focused on a sample using an objective lens (40 × 0.75NA UPlanFLN, Olympus, Tokyo, Japan). The samples were prepared as droplets of the purified fluorescent proteins dissolved in buffered solution (pH 5.5–10.0), applied to a standard 24 × 24 mm cover glass (Heinz Herenz, Hamburg, Germany). The average laser power was tuned with a polarizing attenuator in the range of 0.1–10 µW. The fluorescence (>505 nm) passed back through the objective lens and laser coupling dichroic mirror was directed towards the input of an Acton SP300i monochromator with a hybrid single-photon detector on its output (HPM-100-6, Becker&Hickl, Berlin, Germany), registering the fluorescence decay kinetics in the 510–520 nm band. The fluorescence decay data were analyzed using SPCImage software (Becker&Hickl, Berlin, Germany) with the measured instrument response function (IRF).

#### 3.3.2. Single-Photon 450 nm Fluorescence Excitation

Measurements were made using a time-resolved miniTau fluorescence spectrometer (Edinburgh Instruments, Livingston, UK) in a 50 ns window divided into 2048 time channels. The fluorescence was excited using an EPL-450 picosecond laser (Edinburgh Instruments, Livingston, UK) with a central emission wavelength of 445.6 nm and a repetition rate of 20 MHz. The photons were counted in the spectral range of 475–525 nm. The data processing and visualization and determination of χ^2^ (Pearson’s test) were carried out using the Fluoracle 2.5.1 software (Edinburgh Instruments, Livingston, UK).

#### 3.3.3. Two-Photon 750 nm Fluorescence Excitation

The fluorescence decay data of EGFP-Y145L/S205V were recorded using a single-photon counting (SPC) detector (HPM-100-40C, Becker & Hickl, Berlin, Germany) in the spectral range from 500 to 650 nm, limited by edge pass optical filters (Thorlabs, Newton, MA, USA). An oil immersion objective (PLAN APO 60X/1.4 NA, Nikon, Minato, Japan) was used for the fluorescence excitation. The two-photon fluorescence was excited at 750 nm (repetition rate 80 MHz, pulse width 150 fs, optical power 20 mW) using a femtosecond optical parametric oscillator (TOPOL-1050-C, Avesta Project Ltd., Moscow, Russia) pumped by a Yb femtosecond laser (TEMA-150, Avesta Project Ltd., Moscow, Russia). The post-processing and visualization of the calculated data were performed using Origin Pro 2015 (OriginLab Corporation, Northampton, MA, USA).

### 3.4. Cell Culture, Transfection and Preparation for Microscopy

HEK293T cells (EMBL collection) were seeded onto 35 mm glass-bottomed dishes (MatTek or Fluorodish) and cultured in DMEM with 10% FBS at 37 °C in a 5% CO_2_ atmosphere. After 24 h, the cells were transfected with a mixture of 2 μg DNA and 6 μL FuGene HD transfection reagent in 100 µL of OptiMEM solution per dish. After 12 h, the cell medium was replaced with a fresh DMEM medium.

The preparation of the ionophore-penetrated HEK293T cells expressing EGFP-Y145L/S205V for FLIM scanning was performed as described in [36]. Each pH buffer contained 5 µM nigericin, 5 µM monensin, 130 mM potassium gluconate, 20 mM sodium gluconate, 0.5 mM MgCl_2_, 0.2 mM EGTA and 30 mM Tris (pH 8.0–9.0), HEPES (pH 6.8–7.8) or MES (pH 5.0–6.6). For each pH point, the cell dishes were washed 2 times and incubated for 4 min in a corresponding calibration buffer at 37 °C.

For the monitoring of the stimulated pH fluctuation in the mitochondrial matrix, the HEK293T cells expressing EGFP-Y145L/S205V were treated with 10 μM CCCP.

### 3.5. Fluorescence Lifetime Imaging Microscopy (FLIM) of Live HEK293T Cells

The fluorescence lifetime imaging measurements of the live cultured cells were performed at room temperature in air using an inverted Eclipse Ti-U microscope equipped with a DCS-120 confocal scanning FLIM system and HPM-100-40C hybrid single-photon detector (Becker & Hickl GmbH, Berlin, Germany). An oil immersion objective (PLAN APO 60X/1.4 NA, Nikon, Minato, Japan) was used for the image acquisition. The two-photon fluorescence was excited by pulses at 750 nm obtained using the femtosecond optical parametric oscillator TOPOL (Avesta Ltd., Moscow, Russia) pumped by a Yb femtosecond laser TEMA-150 (Avesta Ltd., Moscow, Russia) and driven by an 80 MHz repetition rate (pulse duration 140 fs). The fluorescence was registered in the range of 500 to 650 nm, limited by edge pass optical filters (Thorlabs, Newton, MA, USA). The image recording time in all cases did not exceed 3 min. The SPCImage software (Becker & Hickl, Berlin, Germany) was used to extract the lifetime distributions from the collected images. The post-processing of the data was performed using Origin Pro 2015 (OriginLab Corporation, Northampton, MA, USA).

## 4. Conclusions

In this contribution, we showed that the emission lifetime of EGFP-Y145L/S205V, a variant of the enhanced green fluorescent protein, shows sensitivity to the pH. This property is better expressed when the fluorescence excitation wavelength is primarily absorbed by a neutral GFP chromophore. Purified EGFP-Y145L/S205V demonstrates a pronounced quasi-linear pH dependence of its lifetime in the pH range of 6–10, which can be plotted based on both the exponentially fitted emission decay and the mean lifetime data (we consider the latter method to be more practical). A similar, albeit significantly alkaline-shifted (from pH 7.0 to 7.5), pH dependence is observed in living cells, allowing us to consider EGFP-Y145L/S205V as a lifetime-based, genetically encoded pH indicator. Such a shift probably reflects the change in the apparent chromophore’s pKa, characteristic of most of the similar probes [17,21,23,26]. We suppose that the probe described here can be successfully visualized under the influences of both single- and two-photon excitation, with the latter being preferable for in-depth tissue imaging and multi-modal microscopy, specifically, combined with alternative fluorescence excitation modes [37] and atomic force microscopy [38,39]. While this probe has obvious drawbacks, such as its short lifetime, which imposes high requirements with respect to the temporal resolution of the signal detection (which, in turn, is strongly determined by the use of advanced hardware, such as light sources with a high repetition rate, a narrow instrument response with an FWHM over a timescale of hundreds of femtoseconds to tens of picoseconds, high-grade TCSPC electronics, etc.) and relatively low photon count, especially at pH < 8.0–8.5, which necessitates a longer acquisition time and demands a lower dark count rate, one can definitely note several important advantages of EGFP-Y145L/S205V, specifically the high dynamic range and near-linear monotonic Tm/pH dependence within the pH range of 7.5–9.0. Such a narrow working pH range limits the application field of the probe but fits well with the concept of a ‘pH paper for a short range’. Indeed, our measurements of the mitochondria show that EGFP-Y145L/S205V can both accurately determine the absolute pH value and detect small pH shifts. The other target organelles for precise pH measurements using this indicator might include the peroxisomes (pH 7.0–8.5, in diverse eukaryotes), cytosol (7.2–7.7), nucleus (7.2–7.4) and endoplasmic reticulum (7.1–7.6) [6]. Moreover, the EGFP-Y145L/S205V indicator could find application in diverse, sophisticated ex vivo and even in vivo models, where the weak alkaline pH needs to be measured in the extracellular or intra-tissual regions [40]. For instance, chronic wounds are characterized by an elevated alkaline milieu (with pH values of up to 8.9) that is considered to affect most of the phenomena involved in wound healing, such as oxygen release, angiogenesis, bacterial toxicity, etc. [41]. Importantly, the accuracy of pH-metry estimated by the FLIM scanning of EGFP-Y145L/S205V can be as high as 0.1 unit.

## Data Availability

The data presented in this study are available within the manuscript and supplementary materials. The raw data are available on request from the corresponding author.

## Supplementary Materials

The following supporting information can be downloaded at: https://www.mdpi.com/article/10.3390/ijms232112907/s1.

## Abbreviations

CCCP	Carbonyl Cyanide m-Chlorophenyl hydrazone
DR	Dynamic Range
EGFP	Enhanced Green Fluorescent Protein
EGTA	Ethylene Glycol-bis(β-aminoethyl ether)-N,N,N′,N′-Tetraacetic Acid
ESPT	Excited State Proton Transfer
ETC	Electron Transport Chain
FLIM	Fluorescence Lifetime Imaging Microscopy
FL	Fluorescence Lifetime
FP	Fluorescent Protein
FWHM	Full Width at Half Maximum
GFP	Green Fluorescent Protein
IRF	Instrument Response Function
PBS	Phosphate Buffered Saline
PCR	Polymerase Chain Reaction
TCSPC	Time-Correlated Single-Photon Counting
