# Supplementary material for: A Single Fluorescent Protein-Based Indicator with a Time-Resolved Fluorescence Readout for Precise pH Measurements in the Alkaline Range
Source: Int J Mol Sci. 2022 Oct 26;23(21):12907. doi: 10.3390/ijms232112907 (PMC9658282; doi:10.3390/ijms232112907)
Supplement: Supplementary file 1 [file ijms-23-12907-s001.zip › ijms-1982746-supplementary.pdf]

Supplementary materials

Single fluorescent protein-based indicator with time-resolved fluorescence readout for precise pH-measurements in alkaline range

T.R. Simonyan, E.A. Protasova, A.V. Mamontova, A.M. Shakhov, K.A. Lukyanov, E.G. Maksimov, A.M. Bogdanov

Supplementary table S1. Fluorescence decay kinetics of EGFP-Y145L/S205V fitted by a biexponential model, recorded at various pH values upon excitation with a 450 nm picosecond and a 490 nm femtosecond laser.

	Ex 450 nm/Ex 490 nm				
pH	τ_1, ps	A_1, %	τ_2, ps	A_2, %	χ^2
5.5	726/736	66/34	3345/2407	34/66	1.30/1.23
6.0	706/731	73/33	3054/2388	27/67	1.45/2.10
6.5	761/730	61/34	3351/2395	39/66	1.25/1.23
7.0	830/720	60/39	3163/2389	40/61	1.13/1.20
7.5	942/736	57/41	3172/2411	43/59	1.19/1.18
8.0	969/746	52/42	2799/2419	48/58	1.21/1.03
8.5	1069/766	53/42	2883/2438	47/58	1.18/1.13
9.0	1103/738	51/42	2778/2419	49/58	1.23/1.10
9.5	1195/750	49/42	2820/2452	51/58	1.21/1.14
10.0	1298/778	47/40	2921/2522	53/60	1.16/1.14

τ is the fluorescence lifetime of the corresponding exponential component; A is a relative contribution (amplitude) of the exponential decay component; χ^2 is the Pearson criterion, which characterizes the goodness of exponential fitting.

(A)

ANOVAOneWay (21.10.2022 13:48:22)

Notes

Input Data

ANOVA

Overall ANOVA

	DF	Sum of Squares	Mean Square	F Value	Prob>F
Model	9	6834.3	759.36667	1.83955	0.12287
Error	20	8256	412.8		
Total	29	15090.3			

Null Hypothesis: The means of all levels are equal.
Alternative Hypothesis: The means of one or more levels are different.
At the 0.05 level, the population means are not significantly different.

Fit Statistics

R-Square	Coeff Var	Root MSE	Data Mean
0.45289	0.02744	20.31748	740.3

Means Comparisons

Homogeneity of Variance Test

Box Charts

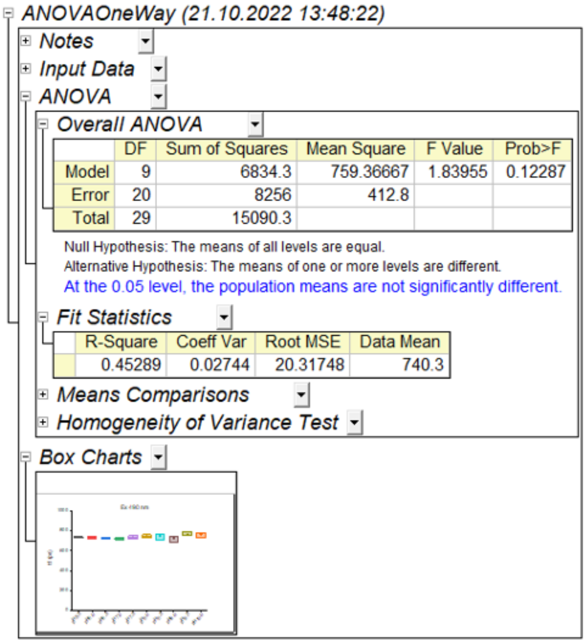

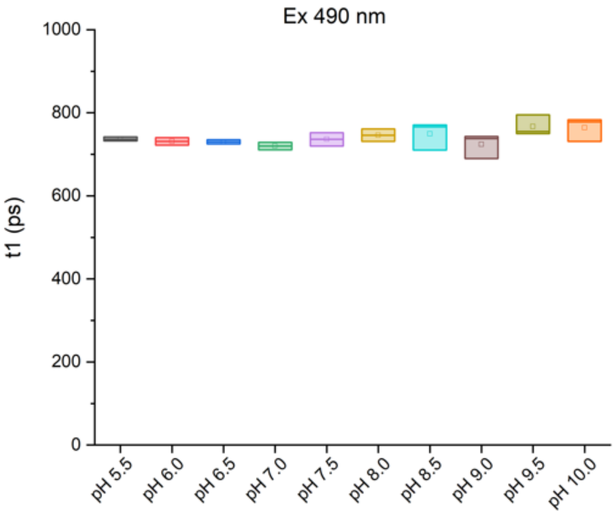

ANOVAOneWay (21.10.2022 14:15:12)

Notes

Input Data

ANOVA

Overall ANOVA

	DF	Sum of Squares	Mean Square	F Value	Prob>F
Model	9	107134.83333	11903.87037	0.77118	0.644
Error	20	308717.33333	15435.86667		
Total	29	415852.16667			

Null Hypothesis: The means of all levels are equal.
Alternative Hypothesis: The means of one or more levels are different.
At the 0.05 level, the population means are not significantly different.

Fit Statistics

R-Square	Coeff Var	Root MSE	Data Mean
0.25763	0.05188	124.24116	2394.83333

Means Comparisons

Homogeneity of Variance Test

Box Charts

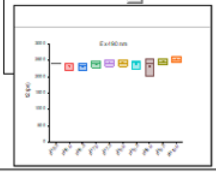

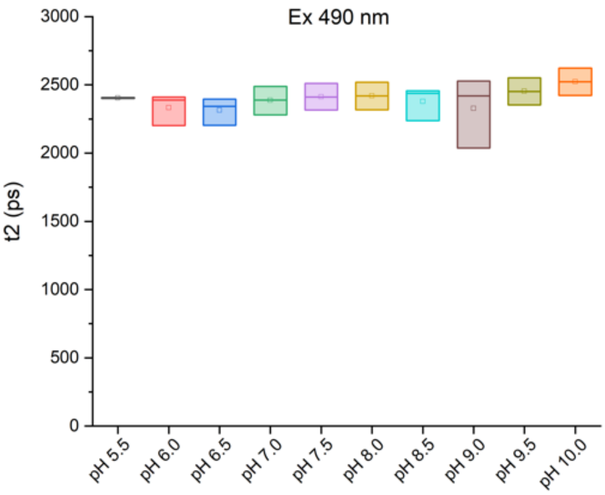

(B)

ANOVAOneWay (21.10.2022 14:30:32)

Notes

Input Data

ANOVA

Overall ANOVA

	DF	Sum of Squares	Mean Square	F Value	Prob>F
Model	9	1146585.46667	127398.38519	48.14754	1.32447E-11
Error	20	52920	2646		
Total	29	1199505.46667			

Null Hypothesis: The means of all levels are equal.

Alternative Hypothesis: The means of one or more levels are different.

At the 0.05 level, the population means are significantly different.

Fit Statistics

R-Square	Coeff Var	Root MSE	Data Mean
0.95588	0.05378	51.43928	956.46667

Means Comparisons

Homogeneity of Variance Test

Box Charts

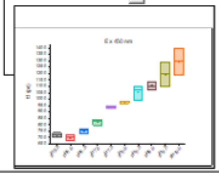

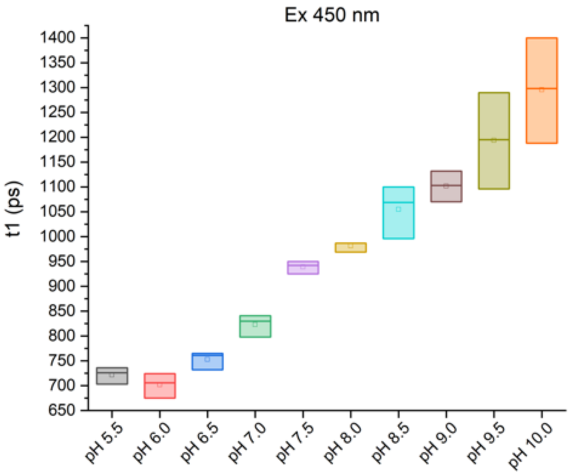

ANOVAOneWay (21.10.2022 14:48:52)

Notes

Input Data

ANOVA

Overall ANOVA

	DF	Sum of Squares	Mean Square	F Value	Prob>F
Model	9	1305348.7	145038.74444	13.18115	1.30751E-6
Error	20	220070	11003.5		
Total	29	1525418.7			

Null Hypothesis: The means of all levels are equal.

Alternative Hypothesis: The means of one or more levels are different.

At the 0.05 level, the population means are significantly different.

Fit Statistics

R-Square	Coeff Var	Root MSE	Data Mean
0.85573	0.03462	104.89757	3029.9

Means Comparisons

Homogeneity of Variance Test

Box Charts

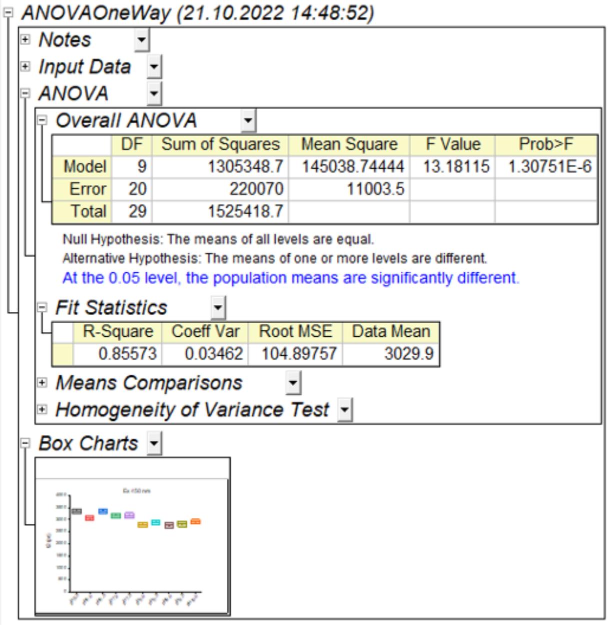

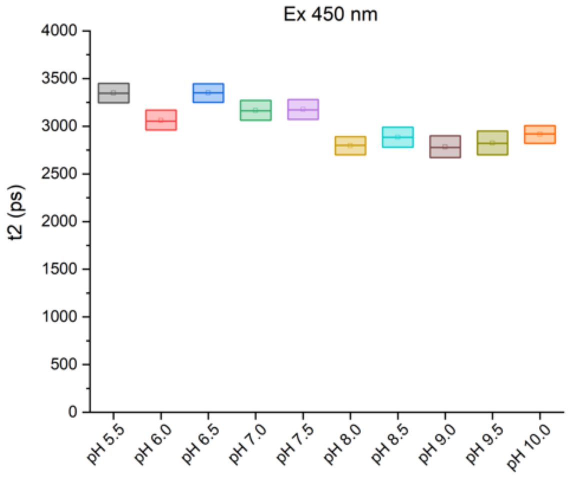

Supplementary Figure S1. Statistical analysis of the fluorescence decay data represented in Fig.1 and Suppl. table 1. Oneway ANOVA test ($p<0.05$) (from the Origin2022b package) was used to analyze the data. (A) Both fluorescent populations (t1 and t2) recorded at 490 nm excitation showed not significant difference. (B) Both fluorescent populations (t1 and t2) recorded at 450 nm excitation showed significant difference.

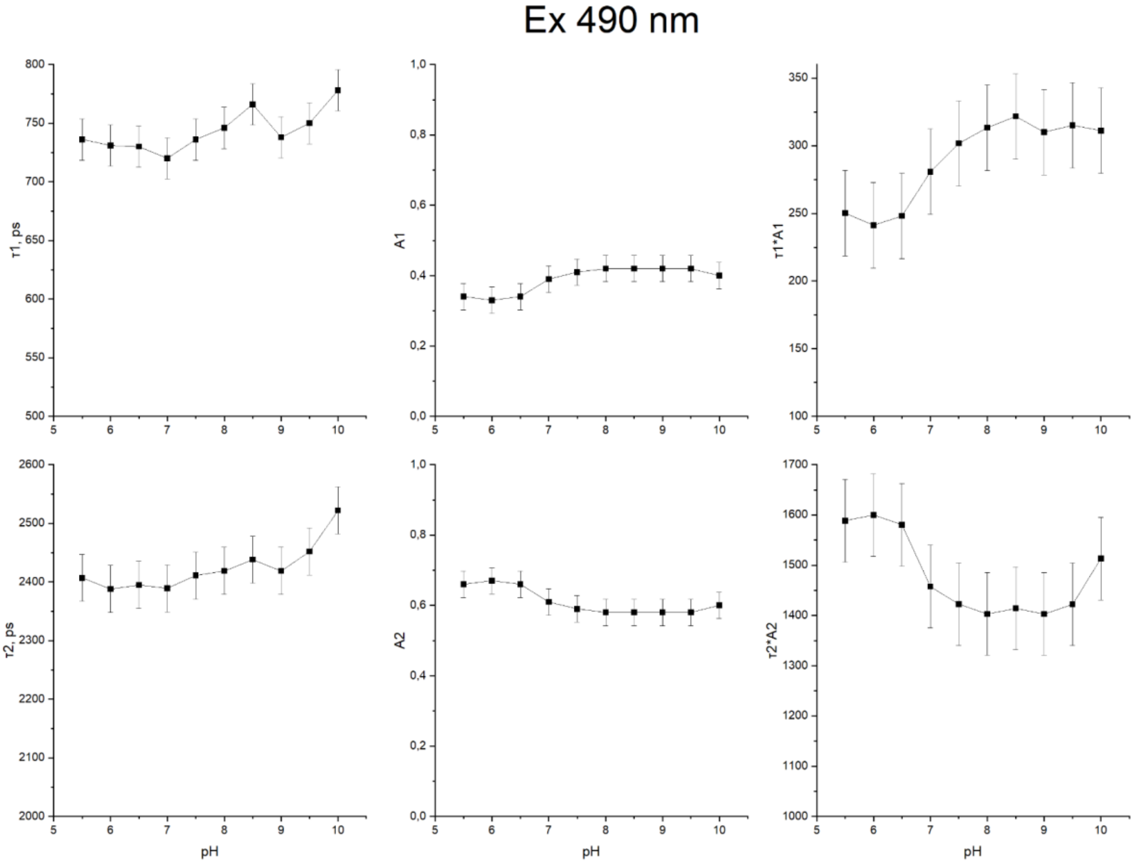

Supplementary figure S2. Fluorescence decay kinetics of EGFP-Y145L/S205V fitted by a biexponential model, recorded at various pH values upon excitation with a 490 nm femtosecond laser. τ is the fluorescence lifetime of the corresponding exponential component (τ1—lifetime of the short-lived component, τ2—lifetime of the long-lived component); A is a relative contribution (amplitude) of the exponential decay component (A1—contribution of the short-lived component, A2—contribution of the long-lived component); τ*A is an amplitude-normalized lifetime (given in a.u.). Standard errors of mean (S.E.M.) are shown for each data point (n = 3).

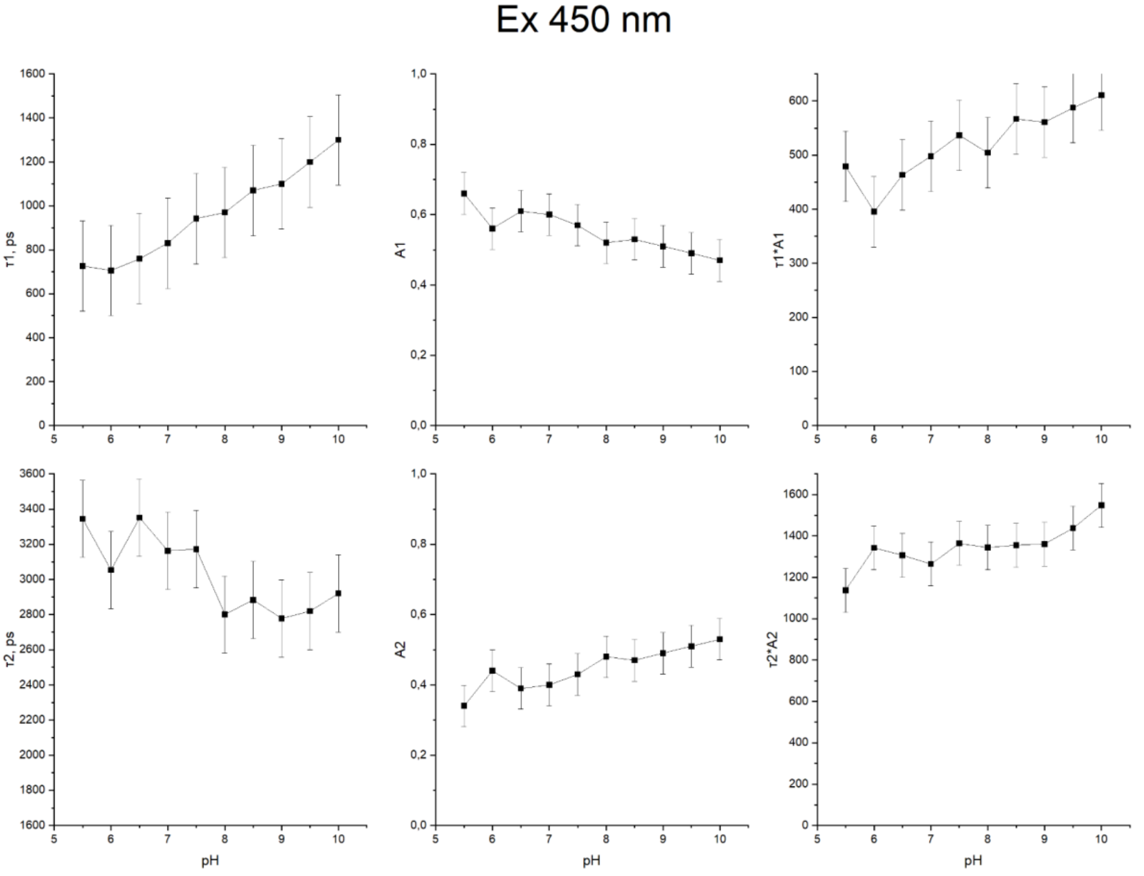

Supplementary figure S3. Fluorescence decay kinetics of EGFP-Y145L/S205V fitted by a biexponential model, recorded at various pH values upon excitation with a 450 nm picosecond laser. τ is the fluorescence lifetime of the corresponding exponential component (τ_1—lifetime of the short-lived component, τ_2—lifetime of the long-lived component); A is a relative contribution (amplitude) of the exponential decay component (A_1—contribution of the short-lived component, A_2—contribution of the long-lived component); τ*A is an amplitude-normalized lifetime (given in a.u.). Standard errors of mean (S.E.M.) are shown for each data point (n = 3).

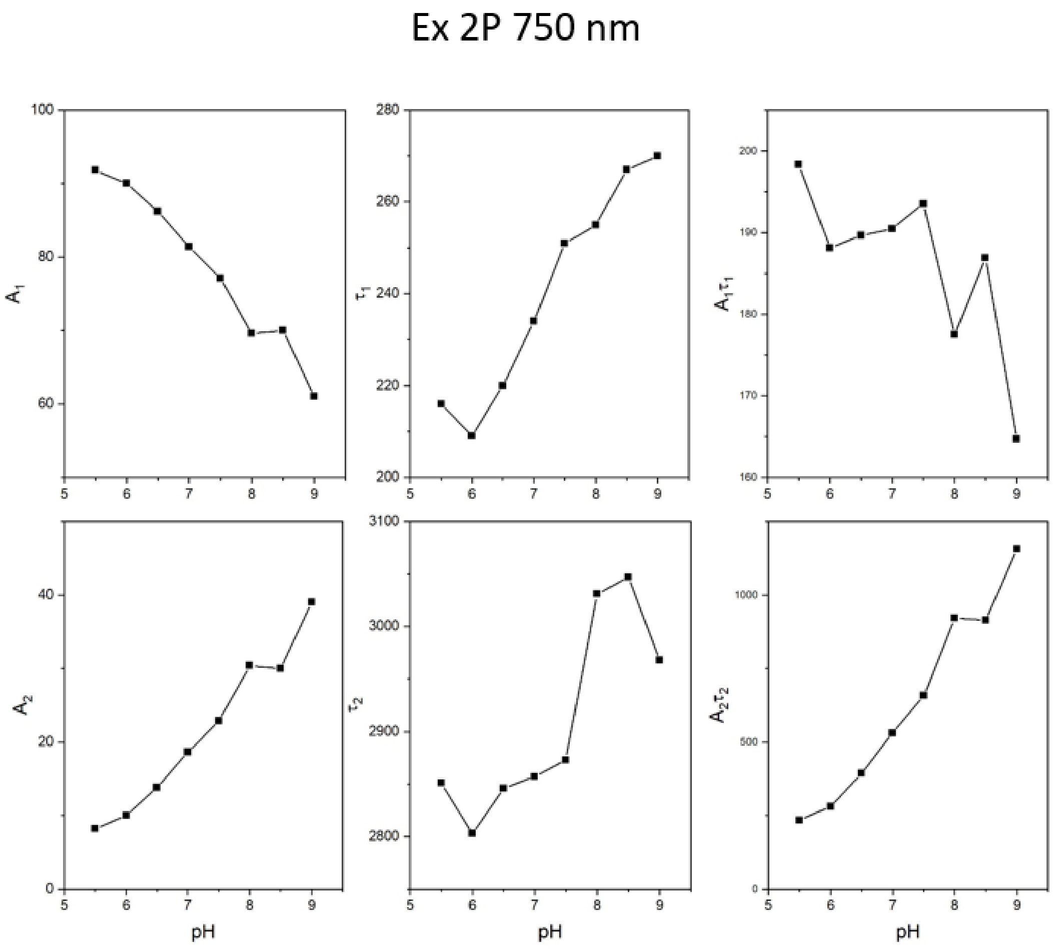

Supplementary figure S4. Fluorescence decay kinetics of EGFP-Y145L/S205V fitted by a biexponential model, recorded at various pH values upon two-photon excitation with a 750 nm femtosecond laser. τ is the fluorescence lifetime of the corresponding exponential component (τ_1—lifetime of the short-lived component, τ_2—lifetime of the long-lived component); A is a relative contribution (amplitude) of the exponential decay component (A_1—contribution of the short-lived component, A_2—contribution of the long-lived component); τ*A is an amplitude-normalized lifetime (given in a.u.).
